# Social Isolation Stress Modulates Pregnancy Outcomes and the Inflammatory Profile of Rat Uterus

**DOI:** 10.3390/ijms23116169

**Published:** 2022-05-31

**Authors:** Nayara A. Lopes, Erin A. Falkenberg, Camille Wiley, Vaishvi Patel, Jesus Serrano-Lomelin, Xin Fang, Amanda M. Weiler, J. Keiko McCreary, Gerlinde A. S. Metz, David M. Olson

**Affiliations:** 1 Department of Obstetrics and Gynecology, University of Alberta, Edmonton, AB T6G 2R3, Canada; nayaraga@ualberta.ca (N.A.L.); cwiley@ualberta.ca (C.W.); vaishvi@ualberta.ca (V.P.); jaserran@ualberta.ca (J.S.-L.); fangxin@ualberta.ca (X.F.); 2 Department of Physiology, University of Alberta, Edmonton, AB T6G 2R3, Canada; 3 Department of Neuroscience, University of Lethbridge, Lethbridge, AB T1K 3M4, Canada; erin.falkenberg@gmail.com (E.A.F.); amandamarie.weiler@gmail.com (A.M.W.); keiko.mccreary2@gmail.com (J.K.M.); 4 Department of Pediatrics, University of Alberta, Edmonton, AB T6G 2R3, Canada

**Keywords:** social isolation, pregnancy, uterus, prenatal stress, preterm birth, gene expression, inflammation, rodents, fetal programming, intrauterine growth restriction

## Abstract

Prenatal stressors have been linked to adverse pregnancy outcomes; including preterm birth (PTB). Recent work demonstrates that social isolation in mothers represents a silent stressor contributing to PTB risk. Here; we investigate the association of inflammatory and stress markers with PTB risk in Long–Evans rats exposed to social isolation stress (SIS) during preconception and pregnancy across four generations (F0-F3). Gestational length; blood glucose; corticosterone levels; and maternal and offspring weights were assessed in two SIS paradigms: transgenerational (TG) and multigenerational (MG) exposure. Maternal uterine tissues were collected 21 days after the dams gave birth. Exposure to SIS reduced pregnancy lengths in the parental generation and neonatal birth weights in the F1 and F2 generations. Interleukin (IL)-1β (*Il1b*) mRNA levels increased in F0 animals but decreased in the offspring of both stress lineages. Protein levels of IL-1β decreased in the TG lineage. Corticotrophin-releasing hormone receptor 1 (*Crhr1*) expression decreased in SIS-exposed F0 animals and increased in the TG-F2 and MG-F1 offspring. Expression of enzyme 11-β hydroxysteroid dehydrogenase-2 (*11bHSD2*) was enhanced in F1 animals. These findings suggest SIS has adverse consequences on the F0 mothers; but their F1–F3 progeny may adapt to this chronic stress; thus supporting the fetal programming hypothesis.

## 1. Introduction

A successful pregnancy requires complex cooperation and interdependence between physiological systems, including neuroendocrine, cardiovascular, and immune systems [1,2]. Environmental stressors may disrupt the homeostatic mechanisms within these systems, leading to pregnancy complications and affecting fetal health and development [3]. Stress is known to activate the hypothalamic-pituitary-adrenal (HPA) axis, increase maternal levels of glucocorticoids (GCs) [4], and upregulate proinflammatory cytokines [5] in a complex and non-linear fashion. When acute and/or chronic stress sustains endocrine and immune system activation earlier than 37 weeks of gestation, the risk of preterm birth (PTB) increases [6,7].

The environment an individual is exposed to in early life may be a determinant for long-term disease risk and phenotypic changes [8]. This process is called fetal programming where the health trajectory of the offspring is impacted by adversity in utero during critical periods of rapid growth and organ development [9,10]. Stress experienced by the mother during pregnancy influences fetal programming, and it contributes to her offspring’s allostatic load (AL). The AL conceptual framework describes the cumulative burden of chronic stress over the course of life resulting in wear and tear on the body [11,12]. Such cumulative effects in the form of AL may dysregulate the inflammatory cytokine milieu predisposing pregnant individuals to complications such as PTB. Thus, the profound consequences of AL might be transmitted across generations promoting adverse pregnancy outcomes and poor health in the offspring [13].

Our previous studies in rat stress models showed that different types of prenatal maternal stress (PNMS) result in various degrees of adverse pregnancy and offspring outcomes [14,15,16]. In our primary series of studies, we introduced a new paradigm whereby a family history of stress programmed physiological and epigenetic pathways that regulate parturition, brain plasticity, and behaviour culminating in shorter gestation of the daughters and granddaughters and delayed growth of the offspring [14,17,18]. This was accomplished using single-hit stress in pregnant rats by forced swimming and restraint in a single generation or across four generations. Given that humans rarely face only a single stress during pregnancy, we further explored the effects of PNMS on pregnancy outcomes using a two-hit stress model combining psychological stressors and intraperitoneal injections of IL-1β [15]. This stress paradigm resulted in increased variation in F0 gestational lengths, affected the growth trajectories of the offspring, increased the occurrence of adverse pregnancy outcomes, and altered uterine markers of stress and inflammation.

The present study presents the next iteration of our PNMS series in which we examined whether social isolation leads to adverse pregnancy and newborn outcomes. Social isolation stress (SIS) is characterized by prolonged lack of social support and interactions which can cause psychological distress, and its prevalence is increasing worldwide [19,20]. Perceived SIS is associated with an increased risk of coronary heart disease and stroke [21], cancer [22], psychiatric disorders [23,24,25], and early mortality [26,27]. When rats are deprived of physical social interactions during SIS rearing, while maintaining regular olfactory, auditory, and visual contact with their counterparts [28,29], they experience mild psychosocial stress [30,31]. Consequences of SIS include functional, morphological, and neurochemical alterations in distinct brain areas, HPA axis changes, disrupted inflammatory responses, and altered behaviour in adult offspring [16,31,32,33,34,35,36]. While the effects of SIS have been reported in the literature, little is known about its effects on pregnancy outcomes. In humans, several studies found an association between SIS and depression [25,37,38]. Heightened inflammatory response [39,40,41] and prenatal cortisol levels (HPA dysfunction) [42,43] are known outcomes in depressed pregnant women. Indeed, antenatal depression has been linked to high rates of PTB [44,45] and preeclampsia [46].

**Figure 1:**
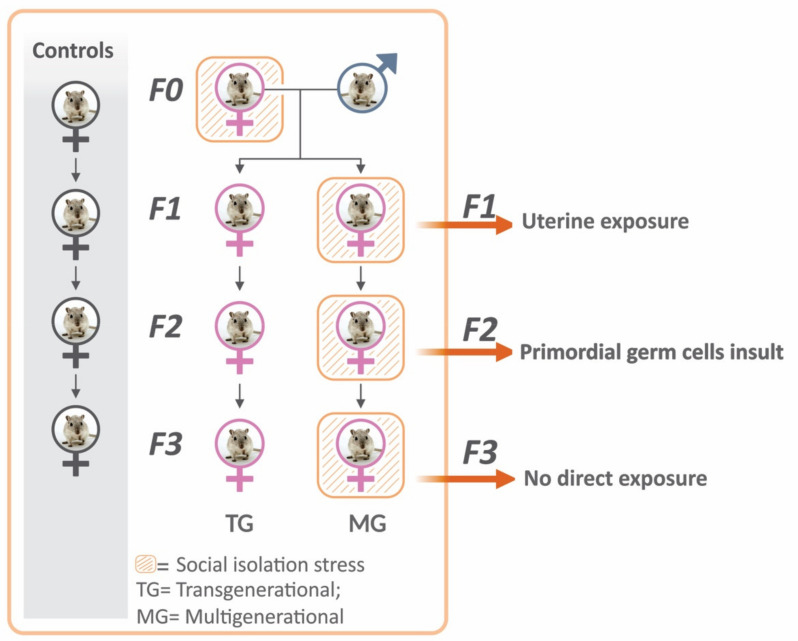
Social isolation stress design. Flow chart illustrating the SIS experimental design. F0 rats were subjected to SIS, and their filial generation F1 was split into TG and MG groups. In the TG group, stress was only implemented in the parental F0 generation, while each parental and offspring generation was exposed in the MG group. F0–F3 generations of non-stressed rats served as controls. In the TG lineage, exposing the gestating female F0 generation to SIS implies that only the F1 fetuses experience direct uterine exposure to stress, and the F2 generation’s exposure to stress is through their mother’s primordial germ cells (F1 generation’s germ cells). Therefore, the F3 generation of the TG lineage is the first unexposed generation, considered a true transgenerational inheritance. In the MG lineage, all generations of offspring experience both direct and cumulative uterine exposure to stress through the maternal primordial germ cells. TG = transgenerational; MG = multigenerational; F = filial generation.

In the present study, we subjected rats to SIS from preconception and during pregnancy to evaluate its effects on pregnancy outcomes and uterine tissue inflammatory profile. Most rodents are social mammals that live in groups and participate in constant social interaction with their conspecifics [25,28]. The impact of SIS was assessed across four generations of rats in a transgenerational (TG) and multigenerational (MG) prenatal stress fashion (Figure 1). Stress only occurred in the F0 dams in the TG lineage, while the F0–F3 generations were exposed to cumulative stress in the MG lineage. Both ancestral and cumulative prenatal stress paradigms have been shown to alter behaviour and produce physiological dysfunctions in the offspring [14,16,31]. We hypothesized that pre-pregnancy and gestational SIS would increase the risk of PTB and low birth weight (LBW) and that uterine markers of stress and inflammation would be upregulated across four generations of rats in both a TG- and MG-dependent manner.

## 2. Results

### 2.1. Social Isolation Stress Shortened Gestational Lengths in F0 Dams

**Figure 2:**
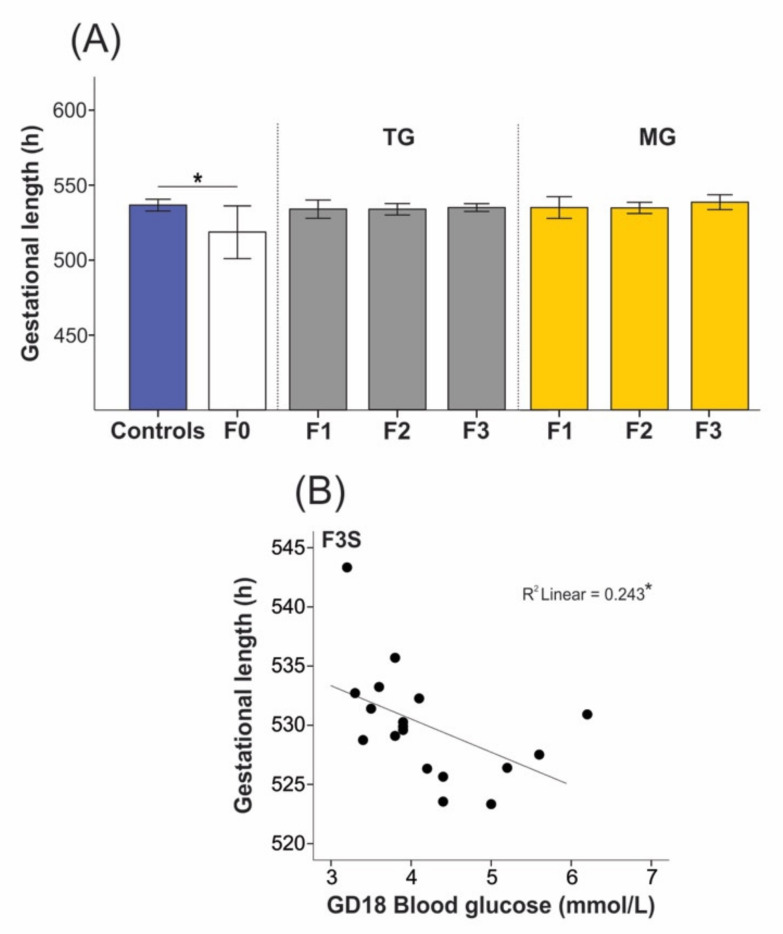
Social isolation stress significantly reduced gestational length of the F0 dams but did not impact the offspring’s pregnancy duration. (**A**) Gestational length recordings demonstrated shorter gestation in the parental generation F0, while no changes were seen in the TG or MG offspring. (**B**) For the F3 generation animals of both TG and MG groups, higher blood glucose levels were associated with shorter gestation in F3 stressed animals on gestational day (GD)18. Asterisks indicate significance: * *p* < 0.05. Controls *n* = 20–28; F0 *n* = 10–11; F1 *n* = 6–8, F2 *n* = 10–12; F3 *n* = 8–11. Mean ± SEM. Independent *t*-test was used to assess gestational length between controls and F0 animals, while Kruskal–Wallis was used to evaluate differences in pregnancy duration between the offspring and controls. TG refers to transgenerational and MG to multigenerational stress.

Gestational lengths were significantly decreased in the F0 stress groups compared to group-housed controls (519.23 ± 24.73 h vs. 530.06 ± 9.26 h, *p* = 0.045; Figure 2A). Pregnancy duration was not reduced in the F1–F3 offspring of the TG group (F1 529.35 ± 7.00 h; F2 528.08 ± 5.78 h; and F3 529.27 ± 3.72 h; *p* = 0.751; Figure 2A) compared to controls. Similarly, gestational length did not change in the F1–F3 offspring of the MG group (F1 527.81 ± 6.53 h; F2 527.59 ± 4.96 h; F3 531.19 ± 5.64 h; *p* = 0.412; Figure 2A). Although the overall gestational length was not affected in SIS-exposed F3 offspring, increased blood glucose levels on gestational day (GD)18 were associated with shorter gestation length (r(16) = −0.493, *p* = 0.038; Figure 2B).

### 2.2. Social Isolation Stress Moderately Impacted the Breeding of Dams

We performed pathological analyses in noticeably unhealthy dams across all groups. Observed adverse health outcomes included disinterest in breeding, inability to become pregnant, pregnancy-related complications, and other health-related adverse events (Table 1). The phi coefficient indicated a moderate relationship between stress and breeding success (φ = 0.326), but the association was not significant (13.5%, *p* = 0.067). The occurrence of health complications such as kidney dysfunction and idiopathic disease was higher in controls (8.1%) compared to stressed (5.4%) animals, although the association was low and not significant (*p* = 0.337, φ = 0.157). Little if any association was observed between treatments (SIS or control) for the variables: inability to become pregnant (*p* = 1.000, φ = 0.023) or pregnancy-related complications (*p* = 1.000, φ = 0.046).

### 2.3. Blood Glucose Levels Were Reduced in the Offspring Exposed to Transgenerational or Multigenerational SIS

**Figure 3:**
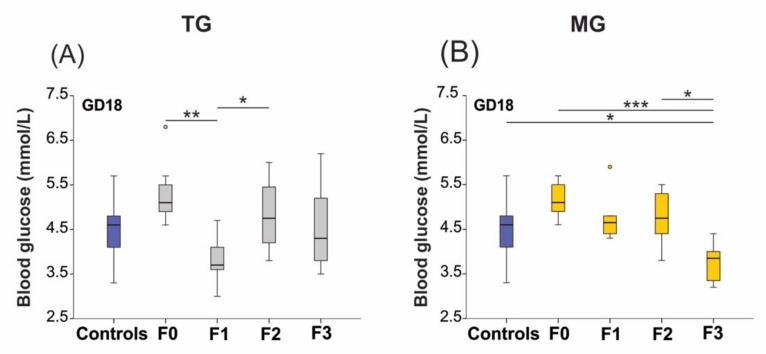
Blood glucose levels decreased on GD18 in the offspring of animals exposed to social isolation prenatal stress. (**A**) Blood glucose levels significantly decreased in F1 animals of the TG group (**A**) and the F3 generation of the MG group (**B**) on GD18. Asterisks indicate significance: * *p* < 0.05; ** *p* < 0.01; *** *p* < 0.001. Controls *n* = 20–28; F0 *n* = 10–11; F1 *n* = 6–8, F2 *n* = 10–12; F3 *n* = 8–11. Box plots mid-lines indicate medians, whiskers indicate min-max values, and boxes indicate interquartile ranges.

Basal glucose levels were unaltered in the TG (*p* = 0.494) and MG (*p* = 0.826) lineages during preconception. However, we observed changes in the SIS animals’ late pregnancy blood glucose concentrations. Blood glucose levels were significantly impacted in the TG (*p* = 0.001) and MG (*p* < 0.0001) stress protocols (Figure 3A,B). In the stressed TG group, glucose levels dropped significantly in the F1 generation (F0–F1, *p* < 0.01) on GD18 (Figure 3A). However, glucose levels returned to baseline in F2 animals (*p* = 0.025). Gestational blood glucose levels were lowest in the F3 generation of the recurrent stress MG group when compared to controls (*p* = 0.047), and the F0 (*p* < 0.0001) and F2 generations (*p* = 0.011) (Figure 3B).

### 2.4. Maternal Weight and Litter Size Were Unchanged by TG and MG SIS 

**Figure 4:**
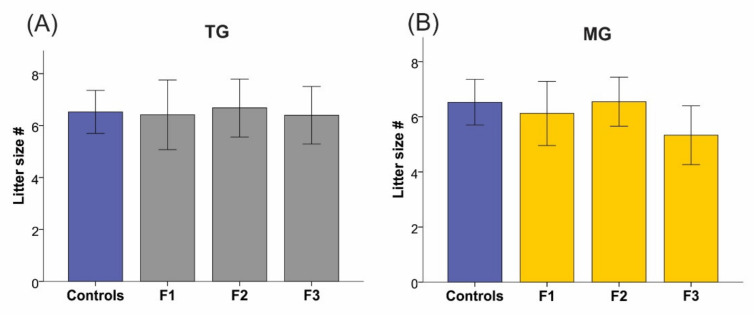
Litter sizes remained unchanged. Litter sizes of dams exposed to SIS did not change in the TG (**A**) and MG (**B**) groups. Controls *n* = 18; F1 *n* = 6–8, F2 *n* = 11; F3 *n* = 9–10. Mean ± SEM.

Maternal (TG *p* = 0.212; MG *p* = 0.372; Table 2) and gestational weights (TG *p* = 0.631; MG *p* = 0.565; Table 2) were unchanged in all groups and generations. Maternal weight gain calculations revealed no changes in either group (TG *p* = 0.189; MG *p* = 0.167; Table 2). Furthermore, no differences in litter size were observed between generations in the TG (*p* = 0.355) and MG (*p* = 0.351) stress lineages (Figure 4). 

### 2.5. Neonatal Growth was Affected in Animals Exposed to SIS, While Plasma Levels of Corticosterone (CORT) Remained Unchanged in the Parental Generation

**Figure 5:**
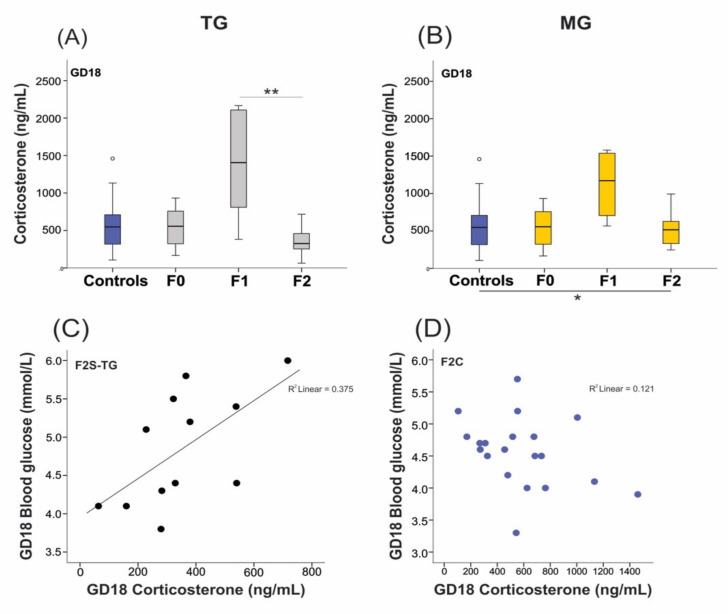
Elevated plasma corticosterone (CORT) levels in F1 animals of TG and MG stress groups. CORT levels were significantly elevated in the F1 dams of the TG (**A**) and MG (**B**) groups on GD18 but returned to baseline levels in the F2 generation. (**C**) Higher CORT levels on GD18 were associated with increased blood glucose levels in the F2 generation of the TG group. (**D**) No correlation was found between CORT and glucose levels in F2 controls. Asterisks indicate significance: * *p* < 0.05; ** *p* < 0.01. Controls *n* = 20–28; F0 *n* = 10–11; F1 *n* = 6–8, F2 *n* = 10–12. Box plots mid-lines indicate medians, whiskers indicate min-max values, and boxes indicate interquartile ranges.

Plasma CORT levels are measured to confirm the presence of stress. Unexpectedly, plasma collected on GD18 displayed basal CORT levels in F0 stressed animals (543 ± 261 ng/mL; Figure 5A) compared to controls (582 ± 332 ng/mL). Levels of CORT were significantly increased in the F1 generation (1399 ±722 ng/mL, *p* = 0.001) of the TG lineage than those of F2 animals (350 ± 179 ng/mL; Figure 5A). The MG lineage demonstrated an overall increase in CORT levels (*p* = 0.037) (Figure 5B), and higher CORT levels were associated with increased blood glucose levels on GD18 in F2 TG-stressed animals (Figure 5C,D).

**Figure 6:**
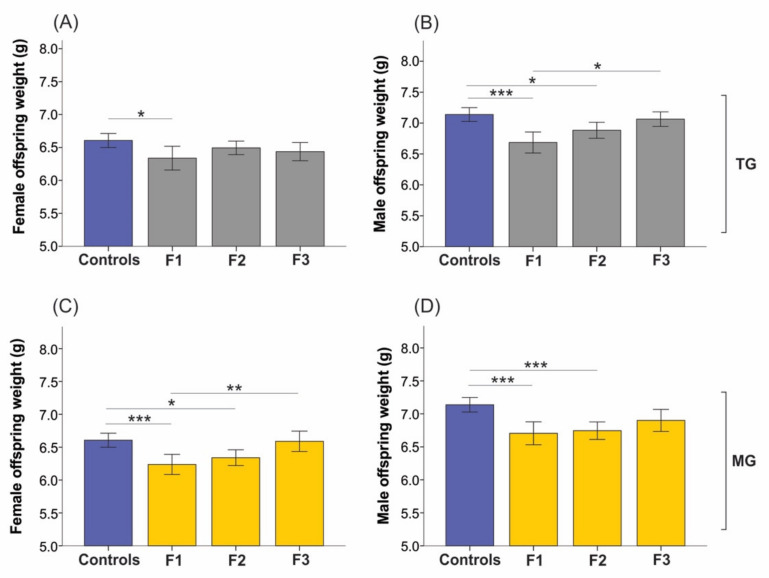
The birth weights of F1 females and males from both SIS lineages were significantly decreased. Birth weights of F1 female (**A**) and F1 and F2 male (**B**) neonates significantly decreased in the TG stress group. (**B**) Yet, F3 TG-stressed males were heavier than F1 male pups. (**C**) Females from the MG group displayed significantly reduced weight on postnatal day (P)1 in the F1 and F2 generations, while F3 females were heavier than F1 stressed animals. (**D**) F1 and F2 MG–stressed males were significantly lighter than controls on P1. Asterisks indicate significance: * *p* < 0.05; ** *p* < 0.01; *** *p* < 0.001. Mean ± SEM. Controls *n* = 115–121; F1 *n* = 37–50, F2 *n* = 68–79; F3 *n* = 47–65 neonates.

Even though CORT amounts in the parental generation were unaltered, the growth of F1 and F2 offspring significantly changed in a sex-specific manner (Figure 6). Daughters (6.34 ± 0.63 g; *p* = 0.019), sons (6.68 ± 0.59 g; *p* < 0.001), and grandsons (6.88 ± 0.57 g; *p* = 0.024) exposed to TG stress were significantly lighter on postnatal day (*p*)1 compared to controls (females 6.61 ± 0.57 g; males 7.14 ± 0.06 g; Figure 6A,B). However, by the F3 generation, TG-male neonate weights normalized to baseline (controls 7.14 ± 0.06 g), and they were significantly heavier than F1 male neonates (7.06 ± 0.46 g vs. 6.68 ± 0.59 g, *p* = 0.019; Figure 6B).

In the cumulative SIS-exposed MG lineage, F1 and F2 females (F1 6.24 ± 0.48 g, *p* = 0.001; F2 6.34 ± 0.50 g, *p* = 0.010) and males (F1 6.70 ± 0.53 g, *p* = 0.001; F2 6.74 ± 0.57 g, *p* < 0.001) displayed significantly lower weight on P1 than controls (Figure 6C,D). Conversely, F3 MG-stressed females were significantly heavier at birth than F1 neonates (6.59 ± 0.54 g vs. 6.24 ± 0.48 g, *p* = 0.009; Figure 6C).

### 2.6. Uterine mRNA Expression of Inflammatory and Stress Response Genes Was Altered in Both SIS TG and MG in a Generation-Dependent Manner

#### 2.6.1. Proinflammatory Cytokines and Their Receptors

**Figure 7:**
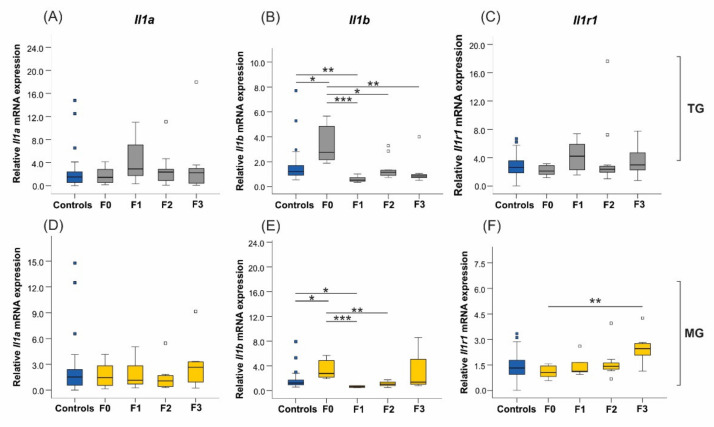
The uterine expression of the *Il1b* proinflammatory cytokine and the *Il1r1* receptor was significantly altered across generations in TG and MG stressed animals. Results from TG and MG groups will be presented alongside each other to compare the effects of stress between the two cohorts. (**A**,**D**) Expression levels of *Il1a* were unaffected in both TG and MG groups. (**B**) The abundance of *Il1b* doubled in the F0 generation and decreased significantly in F1–F3 offspring. Similarly, (**E**) MG-stressed rats presented similar *Il1b* uterine mRNA expression patterns as the TG dams, with significantly increased expression in F0 and a drop in the F1 and F2 generations. The *Il1r1* receptor was significantly upregulated in the F3 uteri of the MG group (**F**), while its expression was unaffected in all generations of the TG group (**C**). Cyclophilin A (*Ppia*) was our reference gene. Asterisks indicate significance: * *p* < 0.05; ** *p* < 0.01; *** *p* < 0.001. Controls *n* = 20–31; F0 *n* = 8–10; F1 *n* = 5–8, F2 + F3 *n* = 4–12. Box plots mid-lines indicate medians, whiskers indicate min-max values, and boxes indicate interquartile ranges.

Uterine expression of the proinflammatory cytokine interleukin-1α (*Il1a*) was not significantly different in either TG (*p* = 0.498) or MG (*p* = 0.744) stress groups (Figure 7A,D). On the contrary, *Il1b* mRNA expression doubled in the stressed F0 generation compared to controls (*p* = 0.026), but then it dropped in the TG F1–F3 offspring as compared to its parental generation (overall *p* < 0.0001; Figure 7B). Similarly, *Il1b* expression in the MG lineage (*p* < 0.0001) displayed a pattern akin to TG stress, except that the differences in F3 animals did not reach significance (F0–F3 *p* = 0.112) (Figure 7E). In the MG group, *Il1b* abundance decreased significantly in the F1 (*p* < 0.0001) and F2 (*p* = 0.003) progeny compared to F0 uteri (Figure 7E). The expression of interleukin-1 receptor 1 (*Il1r1*) significantly increased in the F3 generation of the MG group (F0–F3 *p* = 0.005; Figure 7F), but its expression was unchanged in the TG lineage (*p* = 0.205; Figure 7C). Results for the interleukin-6 (IL-6) proinflammatory cytokine were not included in the analysis, as the mRNA expression in control animals displayed variability within generations.

#### 2.6.2. Corticotrophin-Releasing Hormone (*Crh*) and Its Receptors

**Figure 8:**
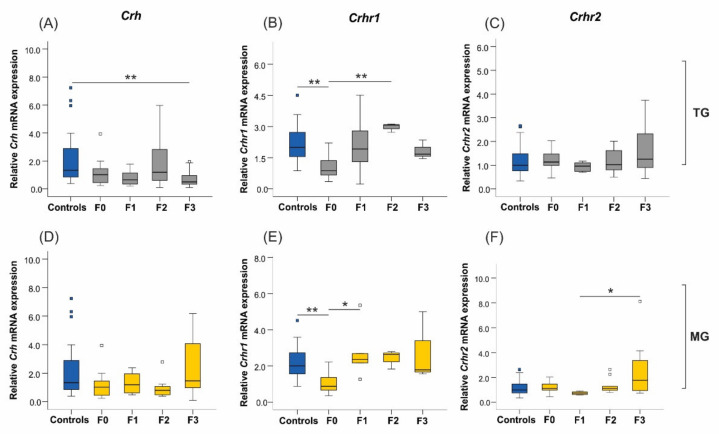
The uterine expression of *Crh* and *Crhr2* reacted differently in TG and MG lineages, while *Crhr1* expression patterns were similar. (**A**) The mRNA expression of *Crh* was significantly reduced in the F3 generation of the TG group, (**D**) yet, no changes were observed in the MG lineage. (**B**) The *Crhr1* expression significantly decreased in F0 animals while its abundance tripled in the TG F2 generation. (**E**) The same pattern was observed in the MG lineage, where uterine expression of *Crhr1* was significantly downregulated in the F0 generation but doubled in the F1 offspring (**C**). The expression of *Crhr2* was unchanged in the TG lineage, while (**F**) it significantly increased in the F3 animals exposed to cumulative MG stress. Asterisks indicate significance. * *p* < 0.05; ** *p* < 0.01. Controls *n* = 20–31; F0 *n* = 8–10; F1 *n* = 5–8, F2 + F3 *n* = 4–12. Box plots mid-lines indicate medians, whiskers indicate min-max values, and boxes indicate interquartile ranges.

There was a significant downregulation of *Crh* expression in the uteri of F3 animals from the TG lineage compared to controls (*p* = 0.007; Figure 8A), but it was unaffected in the MG stress group (*p* = 0.080; Figure 8D). Its receptor, *Crhr1*, was significantly downregulated in the parental generation uteri of the TG (Controls-F0 *p* = 0.007; Figure 8B) and MG (Controls-F0 *p* = 0.007; Figure 8E) stress groups. Although stress exposure differed in the progeny, *Crhr1* expression patterns were similar in both TG and MG lineages. We observed a doubling of *Crhr1* expression in the F1 uteri (F0–F1 *p* = 0.024) exposed to MG stress, whereas its abundance tripled in the F2 uteri of the TG group (F0–F2 *p* = 0.007) when compared to F0 generation. No changes were seen for the corticotrophin-releasing hormone receptor 2 (*Crhr2*) mRNA levels when only F0 animals were socially isolated (*p* = 0.438; Figure 8C), but it was significantly upregulated in the F3 generation of the cumulative stress group (F0–F3 *p* = 0.035; Figure 8F).

#### 2.6.3. 11β-. Hydroxysteroid Dehydrogenase Type 2

**Figure 9:**
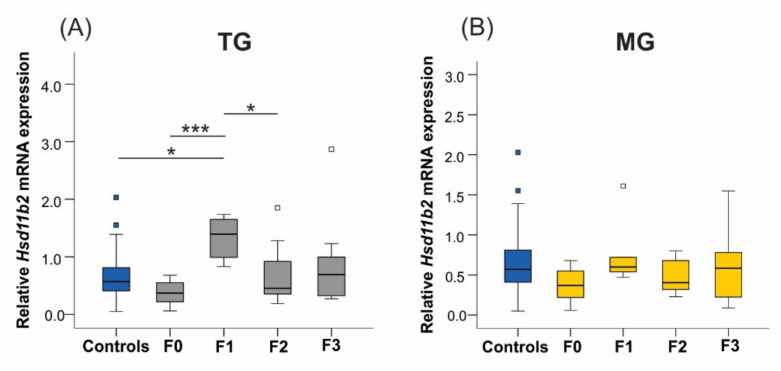
The uterine expression of *Hsd11b2* significantly increased in the F1 generation of the TG lineage. (**A**) The abundance of *Hsd11b2* tripled in the daughters’ uteri. (**B**) Expression levels of *Hsd11b2* were unaltered when exposed to MG SIS. Asterisks indicate significance: * *p* < 0.05; *** *p* < 0.001. Controls *n* = 20–31; F0 *n* = 8–10; F1 *n* = 5–8, F2 + F3 *n* = 4–12. Box plots mid-lines indicate medians, whiskers indicate min-max values, and boxes indicate interquartile ranges.

The 11β-HSD enzyme isoforms 1 and 2 modulate local GC metabolism in the uterus and placenta and regulate the timing of labour [47]. The 11βHsd2 isoform transforms CORT into its inactive dehydrocorticosterone form. In the TG lineage, *Hsd11b2* mRNA levels tripled in F1 daughters (Controls-F1 *p* = 0.023, F0–F1 *p* < 0.0001; Figure 9A) and then normalized in the F2 generation (F1–F2 *p* = 0.036). In contrast, the uterine expression of the *Hsd11b2* gene was unaltered in the MG group (*p* = 0.092; Figure 9B). The *Hsd11b1* results were excluded due to significant variability observed in controls.

### 2.7. Social Isolation Stress Reduces Protein Concentration of IL-1β in F1 Offspring Uteri of Exposed Mothers

**Figure 10:**
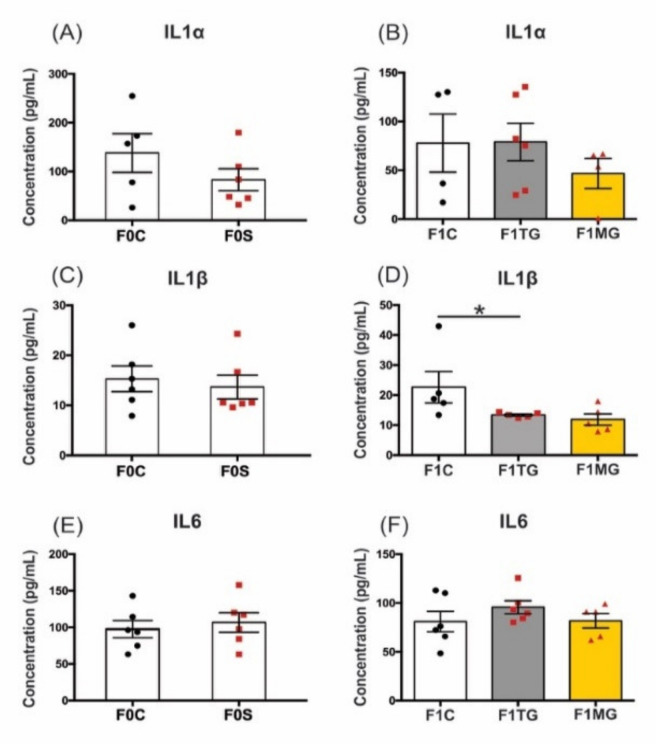
Decreased uterine IL-1β protein concentrations in F1 generation of SIS-exposed dams. (**A**,**B**) The levels of IL1α protein were unaltered in F0 and F1 uteri, although they showed a tendency to decrease in the F1 dams of the MG lineage. (**C**) The IL-1β protein levels in F0 uteri were unchanged, (**D**) but they significantly decreased in the TG lineage compared to F1 controls. In the MG lineage, IL-1β protein also showed a tendency to decrease its levels but did not reach statistical significance. (**E**,**F**) Concentrations of IL-6 were unchanged in both generations and treatment groups. Data are presented as concentration (pg/mL), mean ± SEM. The F0 tissue homogenates were analyzed by independent *t*-test, whereas F1s were analyzed by ordinary one-way ANOVA with Tukey post-hoc test. Statistical significance: * *p* < 0.05, *n* = 4–6.

We performed multiplex protein analysis on three proinflammatory cytokines (IL-1α, IL-1β, and IL-6) related to the onset of labour [48]. Overall, protein concentrations of IL-1α, IL-1β, and IL-6 were unaltered in all F0 treatment groups (F0C–F0S *p* = 0.24; *p* = 0.65; *p* = 0.62, respectively; Figure 10A,C,E). In the F1 generation, however, IL-1β protein levels were significantly reduced in the TG lineage (F(2, 9) = 5.21, *p* = 0.031; Figure 10D) compared to controls (*p* < 0.05). A similar drop occurred in the F1 MG animals, but it was not significant. Conversely, IL-1α and IL-6 protein concentrations did not show significant differences in the F1 generation (F(2, 11) = 0.65, *p* = 0.54 and F(2, 14) = 0.97, *p* = 0.40; Figure 10B,F, respectively). The levels of IL-1α in the MG lineage demonstrated a decrease, but it was not significant (Figure 10B).

## 3. Discussion

In this study, we demonstrated over four generations of rats that PNMS from SIS: (1) induces early birth of the parental generation pups, (2) changes gestational blood glucose levels in the offspring, (3) does not alter maternal gestational weight, (4) leads to increased CORT in the F1 generation, (5) affects neonatal birth weights, and (6) induces fetal programming changes in uterine gene expression of stress and inflammatory markers.

Preterm labour is a complication that remains the leading cause of death during infancy with potentially lifelong neurodevelopmental and chronic consequences [49,50]. Current PTB therapeutics cannot provide a long-lasting delay of PTB and are ineffective in reducing neonatal morbidities [51]. Our primary finding was that SIS reduces pregnancy duration of the parental generation but not enough to cause PTB. This finding is consistent with other stress studies where rodents were exposed to restraint [52], a combination of four stressors [53], and immunological challenges [54]. Pregnant mothers that experienced the Quebec ice storm in 1998 during the first and second trimesters had shorter gestational lengths (38.9 and 38.7 weeks, respectively) compared to those exposed during preconception or the third trimester (39.4 and 39.7 weeks, respectively) [55]. However, in current study, gestation durations normalized in subsequent generations.

The present data revealed a moderate association between SIS and a disinterest in breeding by the dams. There was, however, no significant change in the occurrence of adverse health outcomes in SIS-exposed rats. In contrast, other rodent stress models by our group demonstrated adverse health outcomes such as resorption, preterm, and post-term delivery [14,15] (see Appendix A Table A1 and Table A2). A possible explanation for the differences observed in health outcomes in our studies is the nature of the stress exposure and the number of hits used to stress the animals.

Preconceptional and gestational SIS did not modify the baseline or gestational weights of dams between the groups, and litter sizes remained normal. Previous SIS studies also did not observe differences in body weight [56,57], suggesting some SIS protocols may not be salient to elicit body weight changes in rodents, or differences in strains and species may contribute to these discrepancies.

The SIS study is the third in a series of PNMS studies. We previously subjected pregnant rats to psychological stressors to investigate the programming of physiological and epigenetic pathways by using forced swimming and restraint in a single generation and across four generations—named here single-hit stress [14]. Furthermore, we tested the effects of two-hit stress on pregnant rats’ health outcomes by using a combination of psychological stressors and intraperitoneal injections of IL-1β [15]. A full summary of results and comparisons can be found in Appendix A Table A1 and Table A2.

In all of our PNMS studies, we found that the offspring of stressed rats showed LBWs regardless of the stress type [14,15]. Females and males of both SIS paradigms displayed reduced birth weights in the F1 generation. Sex-specific variations were evident in the F2 generation, where the male birth weights were still low in both paradigms, and female birth weights remained low only in the MG group. These data are consistent with the two-hit stress model [15] and chronic variable mild stress in rats [58]. Birth weights, however, returned to control levels in the F3 generation, indicating a resilience or adaptation to SIS. These findings support the premise that PNMS programs developmental trajectories across generations of offspring in a sex-specific manner.

Exposure to PNMS in a single or over multiple generations of rats has immediate and long-lasting effects on metabolic parameters in the offspring. We observed low blood glucose levels on GD18 in the F1–TG and F3–MG offspring. These findings indicate other layers of regulatory mechanisms on gestational glucose levels in animals exposed to stress. For example, the normalization of blood glucose levels in SIS TG animals after the F1 generation may indicate an adaptation to SIS. Another possible explanation is that stress affects the oscillation of blood glucose levels in rodents [59] which can lead to permanent disruption of glucose metabolism.

We also found an association between higher blood glucose levels and shorter gestation in F3 SIS-exposed animals. This finding agrees with previous observations made in pregnant women [60] and PNMS-exposed rats [14], where elevated glucose levels were associated with shorter pregnancy lengths and altered fetal growth patterns [61]. Increasing CORT levels are associated with elevated blood glucose concentrations in F2–TG animals, suggesting a relationship between higher stress with shorter gestational lengths and higher gestational glucose concentrations. This finding is in accordance with our single-hit stress that only used psychological stressors to expose the rats [14].

Social isolation is considered a mild psychosocial stressor for most rodents and resembles perceived isolation observed in depressive disorders [31,62]. Glucocorticoids (e.g., CORT) and HPA axis mediators are commonly used parameters to evaluate the effects of maternal stress. We did not see changes in plasma CORT in the SIS-treated parental generation, similar to our two-hit stress study [15]. However, unlike the two-hit study, we found elevated CORT levels only in F1 animals in both stress paradigms. Ambeskovic et al. showed that MG psychological stress resulted in HPA axis dysregulation and blunted CORT levels, especially in F4 generation males [63]. Furthermore, absent [64,65] or reduced [66,67] basal CORT levels were reported in rodents due to SIS.

Social stressors in rodents were shown to affect neurobiological mechanisms implicated in depression, including the activation of proinflammatory cytokines, increased levels of GCs, and upregulation of CRH and its receptors [68]. The proinflammatory IL-1β and IL-6 cytokines are key mediators involved in the inflammatory events of parturition [69] and are commonly upregulated in sterile inflammation and intrauterine infection associated with PTB [6,70]. Moreover, the peptide hormone CRH plays a central role in regulating the maternal and fetal HPA axis [71] and exerts its actions by activating two types of receptors, CRHR1 and CRHR2 [72]. Intrauterine CRH and its receptors have crucial roles in parturition [72,73], and their interplay and synergistic effects with proinflammatory cytokines, prostaglandins, and uterine activation proteins regulate the uterus’ transition to a procontractile state [69].

The AL conceptual framework describes how the consequences of recurrent or chronic stress accumulate causing progressive wear and tear on the body. Thus, the concept of AL also applies to the stress passed on to the offspring through the maternal lineage [13]. Observations in all of our PNMS studies (Appendix A Table A1 and Table A2) support a relationship between PTB and transgenerational transmission of stress and inflammatory markers [14,15]. Shorter gestational length in the SIS-exposed F0 animals correlated with high uterine *Il1b* mRNA expression levels. This observation is likely explained by the role IL-1β plays in orchestrating downstream signalling of prolabour mediators [69]. The *Il1b* mRNA expression and protein abundance were reduced in offspring of MG and TG lineages in line with a return to normal gestation lengths. 

The cumulative effects of SIS in the MG group resulted in elevated *Il1r1* receptor abundance in the F3 uteri which may represent a maladaptation. In fact, Ishiguro et al. demonstrated *Il1r1* mRNA upregulation and higher protein abundance before parturition in rats [74]. However, there were no changes in the *Il1r1* and *Il1a* cytokine abundance in uteri of stressed rats from the TG and both stress paradigms, respectively. This intriguing effect may result from an underlying coping mechanism developed by the F1–F3 offspring of dams exposed to chronic SIS, which were revealed as baseline *Il1a* and *Il1r1* receptor expression and decreased Il1b expression in the uteri. 

The actions of IL-1β have been shown to modulate the uterine expression of CRH receptors and their variants and they control the onset of labour [75,76]. The isoform CRHR1 is frequently associated with relaxation of the myometrium, while the isoform CRHR2 is likely involved as a pro-contractile stimulus [72]. Here, we showed a pro-labour profile in the F0 parental uteri, with upregulation of *Il1b* expression and downregulation of *Crhr1*. In the F1–F2 SIS-exposed offspring, the *Crhr1* abundance increased, while the *Crh* mRNA expression downregulated in the F3–TG lineage. These data suggest that the offspring across several generations may be programming uterine gene expression adaptations in response to SIS as a protective and evolutionary mechanism to mitigate future adverse pregnancy outcomes such as PTB.

Cumulative SIS, however, raised *Crhr2* mRNA expression in F3 animals. This change in generational programming would shift the uterus to a prolabour status, thus increasing the risk of PTB in future pregnancies. The offspring PTB protective programming patterns of *Crh* and *Crhr1* versus the pro-PTB patterns of *Crhr2* mRNA expression demonstrate the complex and multifaced functions of CRH and its receptors in regulating various cellular responses and myometrial muscle tone throughout pregnancy. Additional research is warranted to address the cumulative and ancestral effects of stress on the HPA axis and CRH physiological mechanisms in the uterus.

The 11β-Hydroxysteroid dehydrogenase type 2 (*Hsd11β2*) enzyme isoform inactivates CORT into its dehydrocorticosterone form, regulating the fetal exposure to GC and the onset of labour [47]. In the current study, intergenerational transmission of traits was observed in the F1 generation of the TG lineage, with increased *Hsd11b2* expression, while its levels returned to baseline in the F2 generation. This result suggests that higher *Hsd11b2* abundance in the F1 dams is correlated with reduced CORT levels locally in the uterus. This observation further indicates that the offspring are adapting to counteract the effects of SIS. We only assessed the *Hsd11b2* expression in the uterus given that the dams eat their placentas.

Due to the generational nature of this study, we assessed uterine tissues collected on a lactational day (LD) 21 when the pups were weaned. Keeping dams and pups together for 21 days could represent a form of enriched environment and potentially act as a confounding factor for the metabolic and molecular changes observed in the study. However, it was essential to house them together for the proper nutrition and development of the offspring. We would need to perform a separate study to examine the uterine gene expression patterns during parturition. 

Early life is a stage of developmental plasticity where phenotypic changes are influenced by the environment [77]. Our past and present data fit within the match/mismatch and fetal programming hypothesis where early programming of adaptive responses to adversity in anticipation of the postnatal environment occurs in response to stress [77,78,79]. The results of these PNMS studies depict the distinct immediate and generational effects caused by the different stress types [14,15] (Appendix A Table A1 and Table A2).

A common finding among our PNMS studies was LBWs in pups regardless of the stress protocols. This indicates that PNMS directly affects early life outcomes of the progeny and that these effects are transmitted to the future offspring. In humans, LBW is associated with risks for short and long-term complications and disabilities [80,81].

We demonstrated that the expression of key mediators of parturition change in the uteri of stressed animals in all three PNMS studies. These modifications are stress-specific and vary with the duration of the stress protocol [14,15] (Appendix A Table A1 and Table A2). Ultimately, these changes lead to the transmission of inflammatory and stress markers and adverse outcomes over generations of rats thereby increasing future adverse pregnancy outcomes. Prenatal stress was also linked to PTB in humans [7,82] and shown to be passed on to the offspring where it altered the health outcomes of the progeny [83,84].

Our study is timely considering the unprecedented global SIS imposed by the SARS-CoV-2 virus starting in 2020 [25,27,85]. Pregnant women experienced various degrees of isolation during their pregnancies, and a rise in depression and anxiety was observed [86]. Worry, financial pressure, and rates of domestic violence increased while prenatal care visits decreased [87,88,89]. A combination of perceived stress and physiological changes related to infection by the SARS-CoV-2 virus may explain the strong association with increased rates of PTB, preeclampsia, LBW, and gestational diabetes [90]. Indeed, loneliness and social isolation were associated with chronic inflammation during the COVID-19 pandemic [91]. The long-term impacts of the COVID-19 pandemic are expected to be experienced years later, where mothers may leave epigenetic imprints of their stressful experiences on their children, leading to lifelong consequences [92]. 

We found a link between preconceptional and gestational SIS to shorter gestational lengths in the first generation of rats, possibly due to inflammatory imbalance in the uteri. These effects were not observed in the offspring, suggesting the activation of adaptive mechanisms through the generations. Despite the encouraging findings of this study, our society should give cautious attention to this issue, given its possible impacts on pregnancy outcomes of future generations. An integrated multidisciplinary approach and immediate strategies should be implemented worldwide to increase the social support and wellness of pregnant women and reduce the burden of stress and anxiety and build resilience.

## 4. Materials and Methods

### 4.1. Animals and Experimental Design

**Figure 11:**
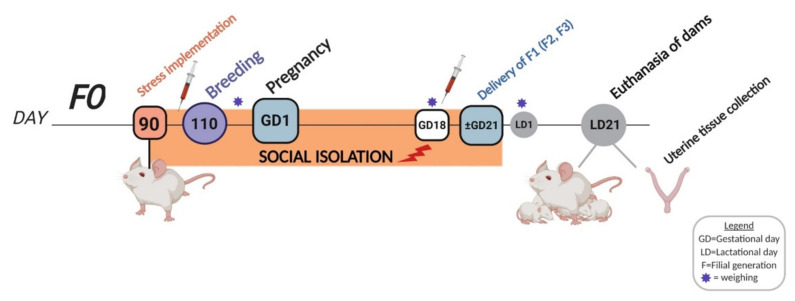
Social isolation stress timeline illustrating the experimental design and tissue collection. Female rats were exposed to SIS during preconception (days 90–110) and pregnancy (GD1–~21). Syringes depict blood draws once at baseline (between days 90–105) and on GD18, while asterisks illustrate weight measurements. Dams were sacrificed at the weaning of their offspring (LD21) when uterine tissues were collected. GD = gestational day; LD= lactational day; F= filial generation. Created with BioRender.com (accessed on 12 May 2022).

Timed-pregnant female Long–Evans hooded rats (*n* = 111) were used to produce a cohort of four generations (F0–F3) of stressed animals. Female rats were individually paired with stress-free males for one hour per day for mating. Breeding was initiated at 110 days of age and continued until pregnancy was detected or females reached 180 days of age (Figure 11). Pregnancy was confirmed by progressive maternal weight gain. Weight measurements were performed on GD18 for dams and P1 for pups. The gestational length was video-recorded from GD20 until birth continuously using an infrared cage site camera (Panasonic WV-BP330, Panasonic, Minato-ku, Tokyo, Japan), and was determined by the total number of hours between final mounting and delivery of the first pup. All pregnant dams were housed individually from GD19 until delivery. The pups were kept with their mothers until weaning (LD21) and then housed with same-sex siblings. Unhealthy dams were excluded from the study and adverse health outcomes were recorded for further analyses.

Females from the parental generation (F0, *n* = 37) were randomly divided into control or SIS groups and bred with stress-free males to produce the filial (F) generation (F1), and their subsequent F2 (granddaughters) and F3 (great-granddaughters) offspring. Timed-Pregnant female rats from the F1–F3 generations were randomly split into transgenerational (TG) or multigenerational (MG) stress lineages (Figure 1). 

Animals were bred and raised locally at the vivarium of the Canadian Centre for Behavioural Neuroscience, University of Lethbridge, AB, Canada. All experiments were conducted in agreement with the Canadian Council on Animal Care and approved by the University of Lethbridge Animal Welfare Committee (Protocol #1715). The experimental procedures were carried out in compliance with the PREPARE guidelines [93] and the results were reported in accordance with the ARRIVE guidelines [94]. All animals were housed in standard cages (45.5 × 25.5 × 20 cm) with the room temperature set at 20 °C and 30% relative humidity. They were subjected to a 12-h light/dark cycle (lights were on at 7:30 and off at 19:30) with ad libitum access to food and water. Litter size was not normalized. Animals were handled the same amount daily throughout the experiments and housed in the same room to control for environmental factors where they could hear and smell their counterparts. 

### 4.2. Pre-Conceptional and Gestational Stress

Psychological SIS consisted of housing a dam alone from P90 for a minimum of two weeks before being paired with a naïve, pair-housed male for breeding (1 h/day). After mating, SIS dams were housed alone until delivery (Figure 11), and they stayed with their pups until LD21. A yoked control group of rats was bred alongside the treatment group, in which animals were housed in pairs throughout the experiment (preconceptionally and during pregnancy) until GD21. 

### 4.3. Tissue Collection

#### 4.3.1. Uteri

In F0, F1 and F2 generations, dams were euthanized at weaning (LD21) via intracardiac injection of Euthanyl (sodium pentobarbital) 300 mg/kg (Bimeda-MTC Animal Health Inc., Cambridge, ON, Canada). In the F3 generation, uteri were collected from virgin females at P90. Following decapitation, uterine horns were extracted immediately and transferred into tubes placed on dry ice, then stored at −80 °C. Left uterine horns were used for gene expression (RT-qPCR) and protein (Multiplex assay) analyses. The number of animals varied between generations and experiments: Controls *n* = 20–31; F0 *n* = 8–10; F1 *n* = 5–8, F2 + F3 *n* = 4–12. 

#### 4.3.2. Blood Sample and Corticosterone Assay

Blood draws (0.5 mL) occurred at baseline prior to social isolation between days 90–105 and on GD18. Blood sampling was performed as previously described by Faraji et al. 2020 [95]. Briefly, blood samples were collected between 9:00 and 11:00 am from the tail vein of animals anesthetized with 4% isoflurane. We isolated plasma by centrifuging the blood samples at 5000 rpm for 5 min. Plasma samples were stored at −80 °C until analyzed for CORT levels using enzyme-linked immunosorbent assay (ELISA) commercial kits (Cayman Chemical, Ann Arbor, MI, USA) as per the manufacturer’s protocol. Non-fasting blood glucose was measured using an Ascensia Breeze Blood Glucose Meter with test strips (Bayer, Mississauga, ON, Canada).

### 4.4. Gene Expression

#### 4.4.1. RNA Extraction

Total RNA was extracted from left uterine horns using Trizol (Thermo Fisher Scientific, Wilmington, DE, USAI) and Qiagen RNeasy Mini Kit on QIAcube (Qiagen, Toronto, ON, Canada) following the manufacturer’s protocol. Total RNA was quantified using NanoDrop ND-1000 spectrophotometer (Thermo Fisher Scientific, Wilmington, DE, USA), and the absorbance ratio 260/280 nm of ~2.0 was accepted as pure for RNA.

#### 4.4.2. Quantitative Real-Time Polymerase Chain Reaction (RT–qPCR)

RT–qPCR was used to quantify genes involved in parturition, inflammation, and stress-related pathways in the uterine horns. Proinflammatory cytokine genes chosen for mRNA expression analysis included *Il1a, Il1b, Il1r1, and Il6* [15,96]. The stress markers selected were *Crh, Crhr1, Crhr2, Hsd11b1* and *Hsd11b2* [73,97].

The reverse transcriptase reaction was performed with total RNA (500 ng) to produce complementary DNA (cDNA) using iScript Reverse Transcription Supermix (Bio-Rad Laboratories, Mississauga, ON, Canada) according to the manufacturer’s protocol. The primers used in this study were previously designed by our group, assuring that the 3′ and 5′ primers spanned over an exon-exon boundary to avoid primers binding to genomic DNA. Primer sequences, annealing temperatures, and accession numbers are described in the Appendix A Table A3. The PCR was completed in duplicates by adding 0.5 μL forward and 0.5 μL reverse primer (10 μM), 10 μL iQ SYBR Green Supermix (Bio-Rad Laboratories, Mississauga, ON, Canada), and 9 μL of cDNA (25 ng/μL) for a total reaction of 20 μL/well. iCycler iQ thermal cyclers (Bio-Rad Laboratories, Mississauga, ON, Canada) were used to run two-step quantitative RT–PCR (amplification and melt curve analysis of nonspecific products) following the protocol: denaturation at 95 °C for 10 min, annealing and elongation for 15 s at 95 °C, and 1 min at the primer-specific annealing temperature (Appendix A Table A3). Repeatability between batches of the same gene experiments was confirmed by preparing a pooled sample with three different cDNA samples combined. This standard sample was included in all PCR plate analyses with proper threshold cycles (Ct) adjustments before data analysis. 

Data analyses were conducted as previously described by Leimert et al. [98]. In brief, cDNA samples were serially diluted to produce a standard curve for each PCR reaction (target genes and the housekeeping gene Ppia) and analyzed with iCycler IQ software (Bio-Rad Laboratories, Mississauga, ON, Canada). The equation E = 10 ^−1/slope^ was used to determine the reaction amplification efficiency using the slope of the standard curve. The average Ct value for each sample was corrected by the efficiency of the reaction. This was repeated for all genes selected in this study. The final threshold cycles were expressed relative to the pooled sample. Target genes data were analyzed according to the Pfaffl method [99] relative to Cyclophilin A (Peptidilprolyl Isomerase A or Ppia) gene expression using the formula:

$$expression\;ratio=\frac{E_{Target}{}^{\Delta Ct\left(Control-Sample\right)}}{E_{Ref}{}^{\Delta Ct\left(Control-Sample\right)}}$$

### 4.5. Luminex Cytokine Assays

Cytokine levels for IL-1α, IL-1β, and IL-6 from F0 and F1 uteri were quantified simultaneously. Analyses were performed using Bio-Plex 200 suspension array system and Bio-Plex 200 software, version 6.0 (Bio-Rad Laboratories, Mississauga, ON, Canada). We utilized Rat Luminex Discovery Assay, a pre-customized magnetic bead-based multiplex assay (R&D Systems, Minneapolis, MN, USA), and followed the manufacturer’s protocol. In brief, left uterine horns (3 mm) were weighted and diluted with 1x phosphate-buffered saline to a concentration of 0.1 mg/mL. Tissues were homogenized using Tissue Lyzer II (Qiagen, Toronto, ON, Canada) with 7 mm stainless steel beads four times for 2 min, 25 Hz cycles. Tissue homogenate protein concentrations were quantified using a BCA Protein Assay kit (Thermo Fisher Scientific, Wilmington, DE, USA) and then immediately stored at −80 °C until use. Multiplex assay was calibrated and validated before sample analyses. Reagents’ preparation and assay were conducted following the manufacturer’s protocol. 

### 4.6. Statistical Analyses

#### 4.6.1. Descriptive Statistics

Animal numbers, means, standard deviations (SD), standard error of the mean (SEM), minimum, and maximum by groups (control and stress) and generations (F0 to F3) for all biological outcomes were calculated and reported previous to inferential analysis (Appendix A Table A4).

#### 4.6.2. Biological Data

Bodyweights, litter size, blood glucose, and CORT were analyzed using the Kruskal–Wallis test (a non-parametric 1-way analysis of variance—ANOVA) with subsequent pairwise comparisons adjusting *p*-values (Bonferroni’s correction). An independent *t*-test was used to assess differences in gestational length between controls and F0 animals, while Kruskal–Wallis test was used to evaluate differences in pregnancy duration between stressed offspring and controls. Pearson correlation coefficient (r) was used to determine the relationships between biological parameters. Fisher’s exact test was used to verify the association between treatment (SIS and controls) and adverse health outcomes (breeding and pregnancy success, health-related outcomes, and pregnancy-related outcomes). The strength of association was assessed by the Phi (φ) coefficient. Gestational length, litter size, and bodyweight data are presented as mean ± SEM.

#### 4.6.3. Molecular Data

Independent-samples median test was performed on the F0–F3 control groups. For each gene, controls that displayed the same median across generations (F0–F3) were pooled (*n* = 20–31). Changes in gene expression across generations were estimated using the Kruskal–Wallis test with subsequent pairwise comparisons adjusting *p*-values (Bonferroni’s correction). TG and MG lineages were analyzed separately. For uterine cytokine abundance using the multiplex immunoassay, data were tested for normal distribution and, when applicable, data were log-transformed to achieve normality. An independent sample *t*-test was used to analyze differences between control and SIS in the F0 generation, while 1-way ANOVA was applied to analyze F1 animals’ data. Significant ANOVA results were further analyzed using Tukey post-hoc testing for multiple comparisons. Levene’s median test was conducted when unequal variances were detected. Data were presented as concentration (pg/mL). Significance was assumed whenever *p* < 0.05. Data coloured in grey represent animals from the TG group, whereas data in yellow display animals from the MG group. All statistical analyses were performed using IBM SPSS Statistics (version 26; IBM Corp, Armonk, NY, USA) and GraphPad Prism (version 5.0; GraphPad Prism, La Jolla, CA, USA).

## Figures and Tables

**Table 1:** Results of the Fisher’s exact test for association between treatment (SIS and controls) and adverse health outcomes. Adverse health outcomes were assessed in noticeably unhealthy dams from both control and stress groups.

Outcomes % (*n*)	Controls (*n* = 15)	Stressed (*n* = 22)	*p*	Strength of Association (φ)
Inability to become pregnant	8.1 (3)	10.8 (4)	1.000	0.023
Health complication	8.1 (3)	5.4 (2)	0.377	0.157
Pregnancy-related complication	2.7 (1)	2.7 (1)	1.000	0.046
Disinterest in breeding	(0)	13.5 (5)	0.067	0.326

**Table 2:** Preconceptional and gestational SIS did not affect maternal weight at baseline or during pregnancy.

		Controls (*n* = 20–28)		F0 Stress (*n* = 10–11)		F1 Stress (*n* = 6–8)		F2 Stress (*n* = 10–12)		F3 stress (*n* = 8–11)		
	Bodyweight (g)	Mean	SD	Mean	SD	Mean	SD	Mean	SD	Mean	SD	*p*-Value
**Transgenerational (TG) group**	Baseline (preconception)	306.87	29.02	322.68	23.50	299.83	22.18	302.37	19.21	312.95	29.41	0.212
	GD18	406.35	34.36	419.86	38.71	396.29	20.20	413.62	25.85	410.21	30.54	0.631
**Multigenerational (MG) group**	Baseline (preconception)	306.87	29.02	322.68	23.50	313.48	24.62	310.27	28.74	306.51	29.96	0.372
	GD18	406.35	34.36	419.86	38.71	401.88	40.70	419.01	33.96	396.85	37.09	0.565

SD: standard deviation. Maternal weight gain is the percent change between baseline and GD18 values.

**Figure A1:**
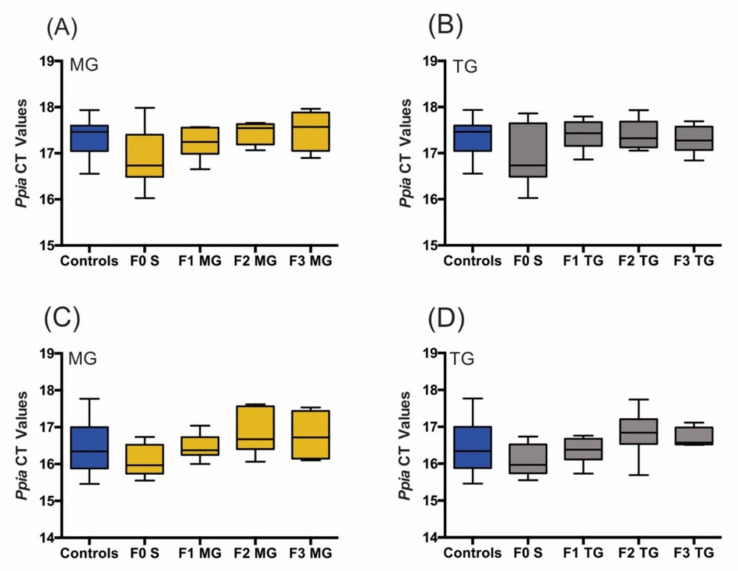
Expression levels of *Ppia* (CT values). No differences were found between generations and stress protocols (TG and MG) using ordinary one-way ANOVA. (**A**,**B**) Batch 1; (**C**,**D**) Batch 2. N numbers: Controls *n* = 20–31; F0 *n* = 8–10; F1 *n* = 5–8, F2 + F3 *n* = 4–12. Mean ± SEM.

## Data Availability

The data presented in this study are available in the article.

## Abbreviations

SIS	social isolation stress
PNMS	prenatal maternal stress
AL	allostatic load
HPA	hypothalamic-pituitary-adrenal axis
GCs	glucocorticoids
CORT	corticosterone
PTB	preterm birth
LBW	low birth weight
TG	transgenerational
MG	multigenerational
IL1-β	interleukin-1β
IL-1α	interleukin-1α
IL1-R1	interleukin-1 receptor 1
IL-6	interleukin-6
CRH	Corticotrophin-releasing hormone
CRHR1	corticotrophin-releasing hormone receptor 1
CRHR2	corticotrophin-releasing hormone receptor 2
HSD11β1	11β-hydroxysteroid dehydrogenase type 1
HSD11β2	11β-hydroxysteroid dehydrogenase type 2
TNF-α	tumour necrosis factor α
LD	lactational day
GD	gestational day
P	postnatal day
F	filial
RT-qPCR	quantitative real-time polymerase chain reaction
Ct	threshold cycles
Ppia	cyclophilin A
SD	standard deviations
SEM	standard error of the mean
ANOVA	analysis of variance

## Appendix A

**Table A1:** Health outcomes comparison between three types of prenatal maternal stress models [14,15].

Outcome	Generations				Stress Model
	F0	F1	F2	F3	
**Gestational length (h)**	▼	ns	ns	N/A	**SIS**
	ns	**▼ TG, MG**	**▼ TG, MG**	N/A	**Single-hit**
	**Increased variation in gestational length**	N/A	-	-	**Two-hit**
**Maternal weight (g)**	ns	ns	ns	ns	**SIS**
	**▼** **GD21**	**▼ MG**	**▼ MG**	N/A	**Single-hit**
	**▼ GD11-18**	N/A	-	-	**Two-hit**
**Pup weight (g) on P1**	N/A	**▼ TG, MG** **♂ ♀**	**▼ MG** **♀** **/TG, MG** **♂**	**▲ MG** **♀** **/TG** **♂**	**SIS**
	N/A	ns	ns	**▼ TG, MG**	**Single-hit**
	N/A	**▼ two-hit** **♂ ♀**	-	-	**Two-hit**
**Blood glucose (mmol/L) on GD18**	ns	**▼ TG**	ns	**▼ MG**	**SIS**
	ns	ns	**▲MG**	ns	**Single-hit**
	ns	ns	-	-	**Two-hit**
**CORT (ng/mL)**	ns	**▲ TG, MG**	ns	-	**SIS**
	ns	ns	**▲MG**	ns	**Single-hit**
	ns	ns	-	-	**Two-hit**
**Adverse health outcomes**	**Moderate disinterest in breeding**				**SIS**
	-				**Single-hit**
	**Resorption, preterm, and post-term delivery**				**Two-hit**

▲: increase; ▼: decrease; ns: non-significant; N/A: not applicable. The dash depicts data not covered in the study. Single-hit stress study: used psychological stressors such as forced swimming and restraint to stress rats in a single generation (TG stress paradigm) or across four generations (MG stress paradigm) [14]. Two-hit stress study (or, Two-stress study): used a combination of psychological stressors and intraperitoneal injections of IL-1β to stress rats [15]. Green colour: data from the current study; off-white colour: data from the single-hit study [14]; grey colour: data from the two-hit study [15].

**Table A2:** Uterine gene expression comparison between SIS and two-hit stress models [15]. Uterine gene expression was not measured in the one-hit stress model of restraint and forced swimming [14].

Gene Expression	Generations				Stress Model
	F0	F1	F2	F3	
***Il1a***	ns	ns	ns	ns	**SIS**
	ns	**▼ two-hit**	-	-	**Two-hit**
***Il1b***	**▲ TG, MG**	**▼ TG, MG**	**▼ TG, MG**	**▼ TG**	**SIS**
	**▲ two-hit**	ns	-	-	**Two-hit**
***Il1r1***	ns	ns	ns	**▲MG**	**SIS**
	ns	ns	-	-	**Two-hit**
***Crh***	ns	ns	ns	**▼ MG**	**SIS**
	▲ two-hit	▼ two-hit	-	-	**Two-hit**
***Crhr1***	**▼ TG, MG**	**▲MG**	**▲TG**	ns	**SIS**
	**▲ two-hit**	**▼ two-hit**	-	-	**Two-hit**
***Crhr2***	ns	ns	ns	**▲MG**	**SIS**
	ns	**trend to ▼ two-hit**	-	-	**Two-hit**
***Hsd11b2***	ns	**▲TG**	ns	ns	**SIS**
	ns	ns	-	-	**Two-hit**

▲: increase; ▼: decrease; ns: non-significant; N/A: not applicable. The dash depicts data not covered in the study. Single-hit stress study: used psychological stressors such as forced swimming and restraint to stress rats in a single generation (TG stress paradigm) or across four generations (MG stress paradigm) [14]. Two-hit stress study (or, Two-stress study): used a combination of psychological stressors and intraperitoneal injections of IL-1β to stress rats [15]. Green colour: data from the current study; off-white colour: data from the single-hit study [14]; grey colour: data from the two-hit study [15].

**Table A3:** Primer forward and reverse sequences and annealing temperatures for RT-qPCR.

Target Gene	Forward Primer (5′ → 3′)	Reverse Primer (5′ → 3′)	Annealing Temperature (°C)	NCBI Reference Sequences *
***Ppia (Cyclophilin A)***	CAC CGT GTT CTT CGA CAT CAC	CCA GTG CTC AGA GCT CGA AAG	60	NM_017101.1
***Crh***	ATCTCACCTTCCACCTTCTG	GTGTGCTAAATGCAGAATCG	60	NM_031019.1
***Crhr1***	GGTGACAGCCGCCTACAATT	AAGGTACACCCCAGCCAA	60	NM_030999.4
***Crhr2***	TGGTGCATACCCTGCCCTAT	GTGGAGGCTCGCAGTTTTGT	60	NM_022714.1
***Hsd11b1***	GAAGAAGCATGGAGGTCAAC	GCAATCAGAGGTTGGGTCAT	60	NM_017080.2
***Hsd11b2***	CGTCACTCAAGGGGACGTAT	AGGGGTATGGCATGTCTCC	55	NM_017081.2
***Il1a***	AAGACAAGCCTGTGTTGCTGAAGG	TCCCAGAAGAAAATGAGGTCGGTC	55	NM_017019.1
***Il1b***	CTCAATGGACAGAACATAAGCC	GGTGTGCCGTCTTTCATCA	51	NM_031512.2
***Il1r1***	CCTGTGATTATGAGCCCACG	CGTGTGCAGTCTCCAGAATATG	58	NM_013123.3
***Il6***	TCCTACCCCAACTTCCAATGCTC	TTGGATGGTXTTGGTCCTTAGCC	65	NM_012589.2

* NCBI—National Center for Biotechnology Information (http://www.ncbi.nlm.nih.gov (accessed on 12 May 2022)).

**Table A4:** Descriptive analysis for biological data. (A) transgenerational group; (B) multigenerational group.

(A)	Transgenerational Group																													
	Control						F0						F1						F2						F3					
	Total N	Mean	SD	SEM	Min	Max	Total N	Mean	SD	SEM	Min	Max	Total N	Mean	SD	SEM	Min	Max	Total N	Mean	SD	SEM	Min	Max	Total N	Mean	SD	SEM	Min	Max
**Gestational Length**	28	530.06	9.27	1.82	520.77	572.8	11	519.24	24.74	7.82	449.4	532.45	8	528.35	7	2.48	511.77	534.27	12	528.09	5.79	1.67	514	536.85	11	529.28	3.73	1.12	523.33	535.7
**Baseline Body weight**	28	306.87	29.02	5.48	256	375.3	11	322.68	23.5	7.08	274.6	347	8	299.83	22.18	7.84	261.4	335.7	12	302.37	19.21	5.55	271.5	332.8	11	312.95	29.41	8.87	269.1	380.1
**GD18 Body weight**	28	406.35	34.36	6.49	354.4	478.9	11	419.85	38.71	11.67	345.8	467.4	8	396.29	20.2	7.14	363.6	422.6	12	413.62	25.85	7.46	377.2	459.5	11	410.21	30.54	9.21	374.7	474.1
**Baseline Blood glucose**	28	7.95	0.96	0.18	5.8	9.7	11	7.95	0.66	0.2	7.3	9.2	8	7.48	0.6	0.21	6.7	8.3	12	7.92	0.98	0.28	6.6	10	11	7.59	0.51	0.15	6.6	8.2
**GD18 Blood glucose**	28	4.54	0.55	0.11	3.3	5.7	11	5.25	0.63	0.2	4.6	6.8	8	3.81	0.52	0.19	3	4.7	12	4.84	0.74	0.21	3.8	6	11	4.54	0.92	0.29	3.5	6.2
**Baseline CORT**	28	886.7	344.19	71.77	412.5	1671.7	11	1027.86	395.13	119.14	496.8	1548.1	8	2022.46	1214.44	429.37	389	3940.6	12	775.27	326.61	94.28	309.3	1450.6	--	--	--	--	--	--
**GD18 CORT**	28	581.56	331.99	74.24	105.6	1459.8	11	543.9	261.17	82.59	166	932.9	8	1398.99	721.76	255.18	382.4	2165.8	12	350.42	178.65	51.57	63.7	716.7	--	--	--	--	--	--
**(B)**	**Multigenerational group**																													
	**Control**						**F0**						**F1**						**F2**						**F3**					
	**Total N**	**Mean**	**SD**	**SEM**	**Min**	**Max**	**Total N**	**Mean**	**SD**	**SEM**	**Min**	**Max**	**Total N**	**Mean**	**SD**	**SEM**	**Min**	**Max**	**Total N**	**Mean**	**SD**	**SEM**	**Min**	**Max**	**Total N**	**Mean**	**SD**	**SEM**	**Min**	**Max**
**Gestational Length**	28	530.06	9.27	1.82	520.77	572.8	11	519.24	24.74	7.82	449.4	532.45	6	527.82	6.54	2.67	518.37	534.78	10	527.59	4.97	1.57	522.07	535.92	8	531.2	5.64	2	523.55	543.33
**Baseline Body weight**	28	306.87	29.02	5.48	256	375.3	11	322.68	23.5	7.08	274.6	347	6	313.48	24.62	10.05	280.5	340.6	10	310.27	28.74	9.09	262.5	338.6	8	306.51	29.96	10.59	266.5	366.6
**GD18 Body weight**	28	406.4	34.4	6.5	354.4	478.9	11	419.9	38.7	11.7	345.8	467.4	6	401.9	40.7	16.6	345.2	448.5	10	419	34	10.7	351.7	470.9	8	396.9	37.1	13.1	337.4	456.2
**Baseline Blood glucose**	28	7.95	0.96	0.18	5.8	9.7	11	7.95	0.66	0.2	7.3	9.2	6	8.28	0.72	0.29	7.6	9.1	10	7.95	0.75	0.24	6.7	9.1	8	7.76	1.08	0.38	6.6	9.6
**GD18 Blood glucose**	28	4.54	0.55	0.11	3.3	5.7	11	5.25	0.63	0.2	4.6	6.8	6	4.78	0.58	0.24	4.3	5.9	10	4.8	0.55	0.17	3.8	5.5	8	3.75	0.42	0.15	3.2	4.4
**Baseline CORT**	28	886.7	344.19	71.77	412.5	1671.7	11	1027.86	395.13	119.14	496.8	1548.1	6	2139.37	1297.8	529.83	892.5	4493.9	10	626.85	309.28	97.8	236.7	1201.1	--	--	--	--	--	--
**GD18 CORT**	28	581.56	331.99	74.24	105.6	1459.8	11	543.9	261.17	82.59	166	932.9	6	1122.57	429.1	175.18	566.6	1580.5	10	527.89	228.9	72.38	246.6	993.6	--	--	--	--	--	--
